# Analysis of QTLs Associated with the Rice Quality Related Gene by Double Haploid Populations

**DOI:** 10.1155/2014/781832

**Published:** 2014-11-12

**Authors:** Gyu-Ho Lee, Byung-Wook Yun, Kyung-Min Kim

**Affiliations:** Division of Plant Biosciences, School of Applied Biosciences, College of Agriculture & Life Science, Kyungpook National University, Daegu 702-701, Republic of Korea

## Abstract

We investigated the growth characteristics and analyzed the physicochemical properties of a doubled haploid population derived from a cross between “Cheongcheong” and “Nagdong” to breed a rice variety that tastes good after cooking and to detect quantitative trait loci (QTLs) associated with the taste of cooked rice. The results showed that these compounds also represent a normal distribution. Correlation analysis of the amylose, protein, and lipid contents indicated that each compound is related to the taste of cooked rice. The QTLs related to amylose content were 4 QTLs, protein content was 2 QTLs, and lipid content was 2 QTLs. Four of the QTLs associated with amylose content were detected on chromosomes 7 and 11. The index of coincidence for the QTLs related to amylose, protein, and lipid content was 70%, respectively. These markers showing high percentage of coincidence can be useful to select desirable lines for rice breeding.

## 1. Introduction

Rice is the main food resource worldwide and the staple food in Korea. With the advent of a new hybridization technique in the late 1960s, the “Tongil” variety was developed by setting up a cross between* indica* and* japonica* rice varieties; up to the 1980s, this variety produced sufficient yield to meet the requirements of South Korea. At present, improved* japonica* varieties are usually cultivated, and the “Tongil” type varieties are only occasionally cultivated for processing and special purposes. Since the 1980s, the demand for high-quality rice has increased due to the diverse requirements of consumers. Therefore, improving the chemical composition and physical characteristics of rice is essential for developing rice varieties according to the preference of consumers and having health benefits as well as good quality [1]. Rice quality parameters such as the appearance, nutrition value, and taste and texture of cooked rice, color, and whole kernel morphology are important factors that affect consumer preferences. In addition, the major chemical components, that is, carbohydrates (72–75%), proteins (7–10%), and lipids (1%), are important factors that reflect the nutritional aspect of rice; the structure and compositions of the proteins and fatty acids are also important parameters. For cooked rice, texture and odor are important factors that determine the quality. In particular, texture is affected by the degree of solidity, adhesion, and cohesion; the ratio of amylose to amylopectin can also affect the texture of cooked rice. Although there are no methods to evaluate objectively the taste because it is a subjective parameter, taste is an important factor to evaluate the quality of rice. Odor is also one of the important factors that affect the taste of cooked rice. The period for which rice is stored after harvest can affect the aroma of cooked rice irrespective of the varieties. Generally, protein and amylose contents determine the taste of rice. Rice that tastes good after cooking is known to have less than 7% protein and 15.5–16.5% water content. Rice with high protein content is hard, less elastic, and less viscous after being cooked. In addition, the unsaturated fatty acid content of rice determines the appearance and quality of cooked rice grains [2, 3]. The crude fatty acid contents of rice include palmitic acid, oleic acid, and linoleic acid. Usually, rice with a high content of lipids is considered unhealthy; however, if the fat content mostly comprises unsaturated fatty acids, the health risks are low.

The QTLs related to amylose, protein, and fat content were detected in previous researches. The QTLs related to amylose and fat content, AMY1 on chromosome 7 and AMY2 on chromosome 8 with 20.4% of phenotypic variation and FAT1 on chromosome 7 with 7.2% of variation, are detected from recombinant inbred lines (RILs) population [4]. Six of QTLs related to amylose content and five QTLs for protein content were detected on chromosomes 2 and 12 [5]. Also rice protein content (RPC) and rice fat content (RFC) were identified from doubled haploid population including two RPC QTLs (qRPC-5, qRPC-7) and two RFC QTLs (qRFC-2, qRFC-5) [6]. Eight QTLs, three QTLs, and six QTLs associated with protein, amylose, and fatty acid, respectively, were detected from RILs population in three different locations which are Wonju, Daegu, and Iksan of Korea [7]. Five QTLs for protein content (PC) were detected on chromosomes 3, 4, 5, 6, and 10, respectively. A major QTL qPC-6 was located near the Wx marker RM190 on the short arm of rice chromosome 6, explaining 19.3% of the phenotypic variance and showing an additive effect of 0.471%. The other four QTLs explained 3.9–10.5% of the phenotypic variance and had additive effects of 0.213–0.343%. Four QTLs for fat content (FC) were detected on chromosomes 3, 5, 6, and 8, respectively. Among these loci, qFC-5 on chromosome 5 was the QTL with the largest effect, explaining 12.9% of the phenotypic variance and showing an additive effect of 0.091% [8]. QTL related to rice lipid content (RLC) with 2.37 of LOD score was identified on chromosome 5 from the high lipid mutant line derived from “Dongjin” by T-DNA insertion [9].

In this study, we investigated the growth characteristics and physicochemical properties of a “Cheongcheong” × “Nagdong” double haploid (CNDH) population and performed the analysis of QTL to attempt to determine quantitative trait loci (QTLs) associated with the taste of cooked rice and breed a new variety that produces rice having good taste.

## 2. Materials and Methods

A DH population consisting of 133 lines derived from a cross between “Cheongcheong” and “Nagdong” was cultivated by the Institute of Crop Breeding, College of Agriculture and Biosciences, Kyungpook National University, at an experimental field of the Kyungpook National University, Gunwi-gun, between 2010 and 2012. The plant materials were sown on April 20, 2012, and transplanted by planting density of 30 × 15 cm with one plant per each line on May 20, 2012. All the CNDH lines were planted with their parents with plants of only one line per planting row, and the planting density was 30 × 15 cm. The amount of fertilizer applied was N : P_2_O_5_ : K_2_O = 9.0 : 4.5 : 5.7 kg/10 a. Phosphate and potassium were used as the main fertilizers, whereas nitric fertilizer was 70% of the main fertilizer used, and 30% of the topdressing fertilizer was used at the tillering stage. Herbicides and insecticides for preventing diseases and pests were used as per the standard cultivation method of rice (The Rural Development Administration, 2000). Excluding the plants of the lines that showed remarkable sterility and separating lines, 70 CNDH lines were used to determine the QTLs associated with the quality of cooked rice.

The growth characteristics of rice plants cultivated at the experimental field of Kyungpook National University, Gunwi, in 2012, were investigated. The following growth characteristics of 5 plants of each line were measured: plant height, length of panicles, the number of spikelets per panicle, and percentage of fertile grains. The amylose, lipid, protein, and starch contents of rice were analyzed using near infrared spectroscopy (NIRS), and the crude fat with fatty acid content was determined by the Bio-Farming and Functional Materials for Foods Regional Innovation Center at the Kyungpook National University, Sangju Campus. NIRS (FOSS NIRSystems 6500; USA) was performed three times after damaged and broken grains and green and red kernels were removed.

QTLs associated with rice quality were analyzed using 217 simple sequence repeat (SSR) markers obtained from the Department of Functional Crop in Rural Development Administration. The WinQTL cartographer (WinQTLcart, version 2.5) was used for the QTL analysis. The composite interval mapping (CIM) was used for whole genome scanning to detect QTLs by using WinQTLcart 2.5 at a threshold log of odds (LOD) of 2.5 [10]. The QTL analysis was performed after the CNDH population was cultivated with their parents, “Cheongcheong” and “Nagdong” for 3 weeks in a green house.

## 3. Results

**Figure 1:**
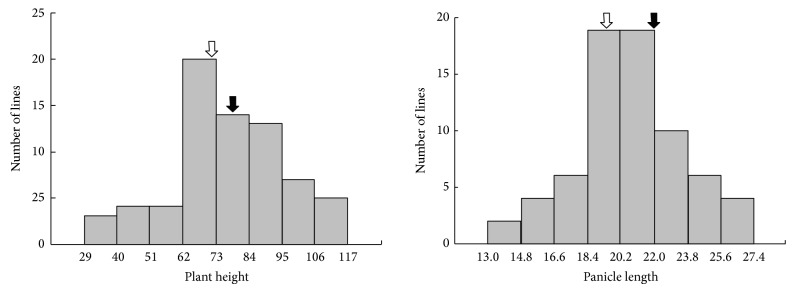
Frequency distributions of the analysis of general growth characteristics of the CNDH population. Black arrow is “Cheongcheong.” White arrow is “Nagdong.”

**Figure 2:**
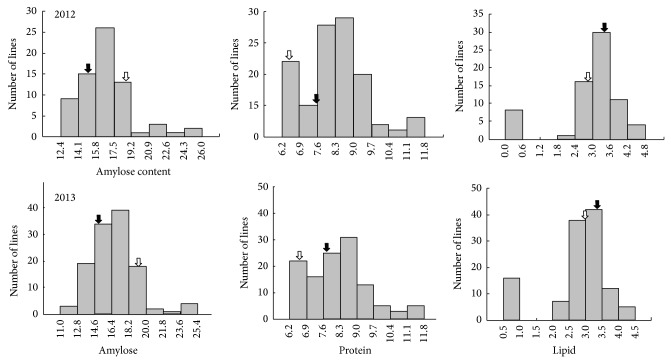
Frequency distribution for the components of rice grains of the CNDH population in 2012 and 2013. Black arrow is “Cheongcheong.” White arrow is “Nagdong.”

Our growth characteristic analysis revealed that the plant height of the CNDH population was lower than that of the parents. Panicle length of the CNDH population was lower than that of “Cheongcheong” and higher than that of “Nagdong.” The number of spikelets per panicle, 1,000 grain weight, and yield of the CNDH population were lower than those of “Cheongcheong” and “Nagdong” (see Supplementary Table 1 in Supplementary Material available online at http://dx.doi.org/10.1155/2014/781832); these parameters showed continuous variation in CNDH, suggesting a normal distribution (Figure 1). Therefore, we analyzed the components of unpolished rice from the CNDH population by using NIRS. The amylose content of “Cheongcheong” is lower than “Nagdong” while the protein and lipid content of “Cheongcheong” is higher than “Nagdong” (Table 1). The correlation coefficient of protein to amylose was 0.280 in the CNDH population. Thus, the correlation analysis revealed that the balance of the three components, amylose, protein, and lipid, is associated with the quality of rice (Table 2). The major 3 factors associated with the taste of cooked rice are generally known as amylose, protein, and lipid. Thus we performed the correlation analysis among these 3 factors in two consecutive years, 2012 and 2013 (Table 2). As shown in Table 2, protein and lipid were significantly different from amylose suggesting that lipid was relatively higher impact factor for determining rice taste. However, in 2013 the factor of protein only seems to be significantly involved in the taste of cooked rice. This slight difference might be due to the differential environmental condition from two consecutive years. Also, the frequency distribution of the three components in the CNDH population showed a similar normal distribution except the frequency distribution in lipid content (Figure 2).

**Figure 3:**
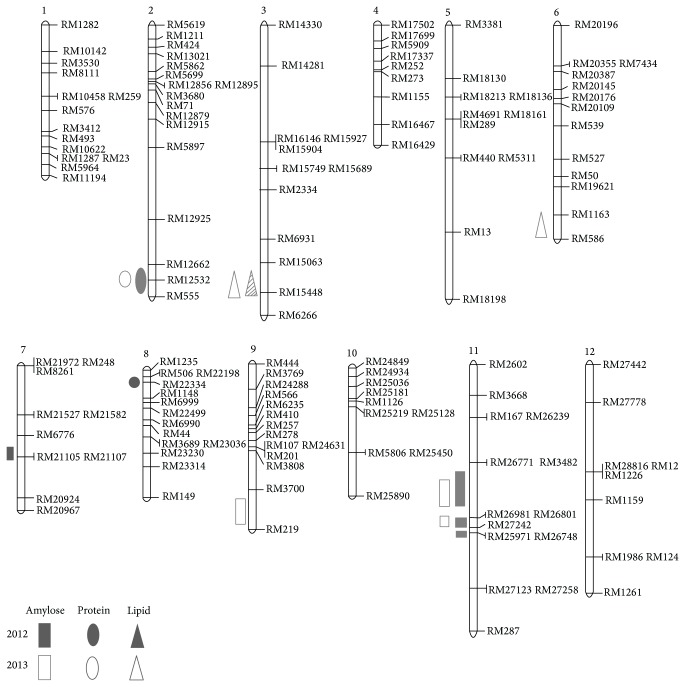
Quantitative trait loci (QTLs) associated with the contents of amylose, protein, and lipid revealed by the genetic map in 2012 and 2013.

The QTLs related to amylose content were qAmy-7, qAmy-11-1, qAmy-11-2, and qAmy-11-3 and that related to protein content was qPro-2. The QTL related to lipid was qLid-3. Four of the QTLs associated with amylose content were detected on chromosomes 7 and 11. RM6776-RM21105 on chromosome 7 had a log of odds (LOD) score of 6.25. RM26771-RM26981, RM26981-RM27242, and RM25971-RM27123 on chromosome 11 had LOD scores of 2.96, 2.58, and 3.59, respectively. The one QTL associated with protein content was detected on chromosome 2; RM12532-RM555 had LOD scores of 4.88. Further, one of the QTLs associated with lipid content was detected on RM15064-RM15448 of chromosome 3 with an LOD score of 3.55 (Table 3, Figure 3). The index of coincidence for the QTLs related to amylose content was 70% for RM21105 on chromosome 7 (Supplementary Table 2) and 80, 75, and 70% for RM26771, RM3482, and RM26801 (Supplementary Table 3), respectively. The index of coincidence for the QTLs related to protein content was 70% for RM12532 and RM555 on chromosome 2 and 75, 75, and 70% for RM506, RM22198, and RM22334 on chromosome 8, respectively (Supplementary Table 4). The index of coincidence for the QTLs related to lipid content was 70% each for RM15063 and RM15448 (Supplementary Table 5) on chromosome 3.

## 4. Discussion

We investigated the growth characteristics and analyzed the physicochemical properties of a doubled haploid population derived from a cross between “Cheongcheong” and “Nagdong” (CNDH) to breed a rice variety that tastes good after cooking and to detect quantitative trait loci (QTLs) associated with the taste of cooked rice. The results suggest that the plant height, panicle length, number of spikelets per panicle, 1,000 grain weight, and yield of the CNDH population show a normal distribution. We further analyzed the contents of amylose, protein, and fatty acids in the rice grains. The results showed that these compounds also represent a normal distribution. Correlation analysis of the amylose, protein, and lipid contents indicated that each compound is related to the taste of cooked rice. In particular, the correlation coefficient of protein to amylose was significant at the level of 0.280. The advent of molecular markers has helped to facilitate the understanding of the genetic behavior of complex quantitative traits. Doubled haploid population derived from “IR64”* indica* type and “Azucena”* japonica* type is exploited for analysis of QTL related to rice quality [11]. This study investigated the growth characteristics and analyzed the physicochemical properties of a doubled haploid population derived from a cross between “Cheongcheong” and “Nagdong” (CNDH) to breed a rice variety that tastes good after cooking and detect quantitative trait loci (QTLs) associated with the taste of cooked rice.

NIRS is known to allow rapid and nondestructive qualitative and quantitative analyses. Because of these advantages, diverse methods such as the development of an automated system by using the NIRS program were attempted to evaluate the taste of cooked rice in developed countries [12]. Despite remarkable development of measurement technology, several factors can affect the result of measurement. In the distribution histogram, the components are not perfect normal distribution because of damage or changing in the content of amylose, protein, and lipid by period and method of harvest, drying, heat, period of storage, and moisture. However, they are similar normal distribution in the tendentious respect.

For determining amylose content and cohesion, alkali digestion value, and protein content, the basic physicochemical characteristics of rice measurement of the changes in amylogram characteristics yield more accurate results [13, 14]. The lower the amylose content, the higher the viscosity of cooked rice. In contrast, the viscosity of cooked rice is low when the amylose content is high because amylose captures amylopectin and inhibits the swelling of starch particles [15]. Also, protein content decreases the taste of cooked rice because a layer of protein develops around the starch particles, reducing the viscosity and elasticity of cooked rice and directly affecting the gelatinization characteristics of starch [16]. The amylose contents between two parental cultivars were shown to be significantly different (Table 1) suggesting differential gene expression of waxy gene, one of the key regulators determining amylose content. Thus, as expected, intermediated extent of amylose content was detected in the hybrid of Cheongcheong × Nagdong double haploid (CNDH) population. Although the smaller amounts of lipids are present in rice than the other components, they are a mediator of heat transfer and impart flavor. Lipids have emulsification characteristics and combine with proteins, thereby affecting the properties of matter in tissues [17].

The QTLs related to amylose content were qAmy-7, qAmy-11-1, qAmy-11-2, and qAmy-11-3 and those related to protein content were qPro-2 and qPro-8. The QTLs related to lipid were qLip-2 and qLid-3. Four of the QTLs associated with amylose content were detected on chromosomes 7 and 11. The index of coincidence for the QTLs related to amylose content was 70% for RM21105 on chromosome 7 and 80, 75, and 70% for RM26771, RM3482, and RM26801, respectively. The index of coincidence for the QTLs related to protein content was 70% for RM12532 and RM555 on chromosome 2 and 75, 75, and 70% for RM506, RM22198, and RM22334 on chromosome 8, respectively. The index of coincidence for the QTLs related to lipid content was 70% each for RM15063 and RM15448 on chromosome 3. Many QTLs associated with rice components have been identified from previous researches. The QTLs for amylose content were detected on chromosomes 3, 6, and 7 commonly [4, 7, 11]. The QTLs for protein content were detected commonly on chromosomes 3, 6, and 7 [6–8]. Also the QTLs for lipid content were commonly detected on chromosomes 5, 6, and 7 [6–9]. In spite of many results, several new QTLs related to amylose and proteins were detected on chromosomes 11 and 2, respectively. The QTLs for lipid on chromosome 3 were identified by Yu et al. [8]. Therefore, we can exploit several SSR markers on chromosomes 11 and 2 showing high coincidence ratio between genotype and phenotype. The markers can be useful to select desirable lines for rice breeding and facilitate further research.

## Supplementary Material

Supplementary Table 1 in analysis of the growth characteristics at DH population.
Supplementary Table 2 in high level of amylose content.
Supplementary Table 3 in low level of amylose content.
Supplementary Table 4 in low level of protein content.
Supplementary Table 5 in low level of lipid content.

## Figures and Tables

**Table 1:** Comparative analysis of the main components of rice grains of the CNDH population with those of the parents, “Cheongcheong” and “Nagdong.”

Year	Item	Amylose	Protein	Lipid
2012	Cheongcheong	15.3 ± 2.1^a^	7.7 ± 1.8	3.4 ± 0.8
	Nagdong	18.4 ± 3.6	6.4 ± 2.4	2.9 ± 1.2
	CNDH	16.7 ± 2.6	8.3 ± 1.2	2.9 ± 1.2

2013	Cheongcheong	15.1 ± 2.8	7.8 ± 1.2	3.5 ± 1.1
	Nagdong	18.8 ± 2.7	6.3 ± 2.7	3.0 ± 1.2
	CNDH	16.8 ± 2.4	8.2 ± 1.3	3.1 ± 0.6

^a^Mean ± standard deviation.

**Table 2:** Analysis of the correlation coefficients for amylose, protein, and lipid.

Year	Components	Amylose	Protein	Lipid
2012	Amylose	1	0.280^*^	0.371^**^
	Protein		1	0.108
	Lipid			1

2013	Amylose	1	0.326^*^	0.163
	Protein		1	0.010
	Lipid			1

^*^Significant at the level of 0.05, ^**^significant at the level of 0.01.

**Table 3:** Analysis of quantitative trait loci (QTLs) associated with amylose, protein, and lipid.

Year	Traits	QTL	Marker interval	Chromosome	LOD^a^	Variance (%)	Additive effect
2012	Amylose	qAmy-7	RM6776–RM21105	7	2.65	33	1.3
		qAmy-11-1	RM26771–RM26981	11	2.96	51	1.9
		qAmy-11-2	RM26981–RM27242	11	2.58	27	1.2
		qAmy-11-3	RM25971–RM27123	11	3.59	32	1.3
	Protein	qPro-2	RM12532–RM555	2	4.88	41	0.6
	Lipid	qLip-3	RM15063–RM15448	3	3.55	52	0.2

2013	Amylose	qAmy-9	RM219–RM23914	9	2.88	34	0.7
		qAmy-11-1	RM26771–RM26981	11	2.66	40	0.4
		qAmy-11-2	RM26981–RM27242	11	6.13	40	0.6
	Protein	qPro-2	RM12532–RM555	2	2.57	39	−0.3
	Lipid	qLip-3	RM15448–RM6266	3	2.78	30	0.2
		qLIp-6	RM586–RM1163	6	2.70	30	0.2

^a^LOD: log of odds.
