# Supplementary material for: Analysis of QTLs Associated with the Rice Quality Related Gene by Double Haploid Populations
Source: Int J Genomics. 2014 Nov 12;2014:781832. doi: 10.1155/2014/781832 (PMC4247976; doi:10.1155/2014/781832)
Supplement: Supplementary file 1 — Supplementary Table 1 in analysis of the growth characteristics at DH population. Supplementary Table 2 in high level of amylose content. Supplementary Table 3 in low level of amylose content. Supplementary Table 4 in low level of protein content. Supplementary Table 5 in low level of lipid content. [file 781832.f1.pdf]

Appendix A. Supplementary data

Analysis of QTLs associated with the rice quality related gene by double haploid populations

Gyu-Ho Lee, Byung-Wook Yun, Kyung-Min Kim*

Division of Plant Biosciences, School of Applied Biosciences, College of Agriculture & Life Science, Kyungpook National University, Daegu 702-701, South Korea

*Corresponding author: Kyung-Min Kim, E-mail: kkm@knu.ac.kr, Tel: +82-53-950-5711, Fax: +82-53-958-6880

Gyu-Ho Lee, bllizard@knu.ac.kr ; Byung-Wook Yun, bwyun@knu.ac.kr

Supplementary Table 1

Analysis of the general growth characteristics between parents and the double hybridization (DH) population.

Item	Plant height (cm)	Panicle length (cm)	Spikelets per panicle (no.)	Weight of 1,000 grains (g)	Yield (kg/10 a)
Cheongcheong	78.1 ± 3.4[a]	22.2 ± 1.0	14.6 ± 1.6	24.6	611.2
Nagdong	72.1 ± 7.7	19.1 ± 1.0	13.9 ± 2.3	24.2	650.6
CNDH	76.5 ± 19.1	20.6 ± 2.8	14.5 ± 3.1	23.4	498.4

[a]The data are presented as mean ± standard deviation

Supplementary Table 2

The 20 lines having high level of amylose content.

Sample[a]	Parents		CNDH lines with high amylose content																				Index of coincidence (%)
	C	N	49	50	51	65	39	57	67	5	20	19	2	36	55	64	15	27	1	59	56	46	
Content[b]	15.30	18.37	25.36	24.63	23.89	22.42	21.31	21.17	19.28	18.96	18.83	18.50	18.45	18.32	18.10	18.05	17.88	17.84	17.82	17.65	17.61	17.51	
RM21105[c]	O[d]	X[e]	O	O	O	X	X	X	O	O	O	O	X	O	O	O	X	O	X	O	O	O	70%

[a] The parents ‘Cheongcheong’ and ‘Nagdong’ and 20 lines of CNDH population with high amylose content are used, [b] Amylose content is measured by using NIRS, [c] RM21105 on chromosome 7 is used for PCR, [d, e] The genotypes mean as genotype of Cheongcheong and Nagdong.

Supplementary Table 3

The 20 lines having low level of amylose content.

Sample[a]	Parents		CNDH lines with low amylose content																				Index of coincidence (%)
	C	N	8	16	31	24	58	11	12	52	10	53	3	13	34	26	47	48	4	61	25	9	
Content[b]	15.30	18.37	12.41	12.44	13.14	13.57	13.60	13.66	13.66	13.74	13.91	14.16	14.21	14.27	14.27	14.30	14.40	14.54	14.68	14.72	15.07	15.16	
RM26771[c]	O[d]	X[e]	O	O	O	O	X	O	O	O	O	O	O	X	O	O	X	O	O	O	X	O	80%
RM3482	O	X	X	O	O	O	O	O	O	O	O	O	X	X	O	O	-	O	O	O	O	O	75%
RM26801	O	X	O	O	O	O	X	O	X	X	O	O	O	O	O	X	O	X	O	O	X	O	70%

[a] The parents 'Cheongcheong' and 'Nagdong' and 20 lines of CNDH population with high amylose content are selected, [b] Amylose content is measured by using NIRS, [c] The markers on chromosome 11 are used for PCR, [d, e] The genotypes mean as genotype of Cheongcheong and Nagdong.

Supplementary Table 4

The 20 lines having low level of protein content.

Chr.	Sample[a]	CNDH lines with low protein content																						Index of coincidence (%)
		C	N	60	19	44	2	55	14	59	1	37	54	67	61	56	62	36	5	41	68	5	64	
	Content[b]	7.656	6.381	6.279	6.362	6.365	6.371	6.488	6.611	6.622	6.638	6.758	6.798	6.853	6.880	6.914	6.973	6.981	7.401	7.552	7.631	7.644	7.652	
2	RM12532[c]	O[d]	X[e]	X	O	X	O	X	X	X	X	O	X	X	X	X	X	X	X	X	X	X	X	70%
	RM555	O	X	X	O	X	O	X	O	X	X	O	X	X	X	X	X	X	X	X	X	O	X	70%
8	RM506	O	X	O	O	O	X	X	X	X	X	X	O	X	O	X	X	X	X	X	X	X	X	75%
	RM22198	O	X	O	O	O	X	X	X	X	X	X	O	X	O	X	X	X	X	X	X	X	X	75%
	RM22334	O	X	O	O	O	X	X	X	X	X	X	O	X	O	X	X	X	X	X	X	X	O	70%

[a] The parents 'Cheongcheong' and 'Nagdong' and 20 lines of CNDH population with high amylose content are selected, [b] Amylose content is measured by using NIRS, [c] The SSR markers on chromosome 2 are used for PCR, [d, e] The genotypes mean as genotype of Cheongcheong and Nagdong.

Supplementary Table 5

The 20 lines having low level of lipid content.

Sample[a]	Parents		CNDH lines with low protein content																				Index of coincidence (%)
	C	N	18	55	49	67	56	63	43	44	54	40	60	66	27	50	45	61	68	39	51	47	
Content[b]	3.414	2.934	2.178	2.496	2.531	2.578	2.619	2.620	2.671	2.672	2.675	2.724	2.731	2.776	2.806	2.822	2.849	2.861	2.930	3.002	3.066	3.078	
RM15063[c]	O[d]	X[e]	O	O	O	X	O	O	O	O	O	X	O	O	O	X	X	O	X	X	O	O	70%
RM15448	O	X	O	O	O	O	O	O	O	O	O	X	O	O	O	O	X	O	X	X	X	X	70%

[a]The parents 'Cheongcheong' and 'Nagdong' and 20 lines of CNDH population with high amylose content are selected, [b]Amylose content is measured by using NIRS, [c]The SSR markers on chromosome 3 are used for PCR, [d, e]The genotypes mean as genotype of Cheongcheong and Nagdong.
